# Molecular Characterization of Emerging and Uncommon Enteroviruses C104, C105, and C109 in Respiratory Samples from Maryland, USA, 2018–2024

**DOI:** 10.3390/v17091183

**Published:** 2025-08-29

**Authors:** Amary Fall, Ting X. Zhuang, Alaina Dodge, Omar Abdullah, Julie M. Norton, David Villafuerte, Andrew Pekosz, Eili Klein, Heba H. Mostafa

**Affiliations:** 1 Johns Hopkins School of Medicine, Department of Pathology, Division of Medical Microbiology, Meyer B-121, 600 N. Wolfe St., Baltimore, MD 21287, USA; afall2@jhmi.edu (A.F.); tzhuang2@jh.edu (T.X.Z.); adodge2@jhu.edu (A.D.); oabdull1@jhmi.edu (O.A.); jnorto19@jhmi.edu (J.M.N.); dvillaf2@jh.edu (D.V.); 2 W. Harry Feinstone Department of Molecular Microbiology and Immunology, The Johns Hopkins Bloomberg School of Public Health, Baltimore, MD 21287, USA; apekosz1@jhu.edu; 3 Department of Emergency Medicine, Johns Hopkins School of Medicine, Baltimore, MD 21287, USA; eklein@jhu.edu; 4 Center for Disease Dynamics, Economics and Policy, Washington, DC 20005, USA

**Keywords:** enterovirus C, EV-C105, EV-C104, EV-C109, whole-genome sequencing, immunocompromised host, respiratory infection

## Abstract

**Background**: While enteroviruses (EVs) are recognized causes of diverse illnesses, little is known about the epidemiology and molecular characteristics of uncommon enterovirus C (EV-C) types, including EV-C104, EV-C105, and EV-C109. **Methods**: We conducted genomic surveillance of EVs at the Johns Hopkins Health System between 2018 and 2024 (a total of 3715 samples), identifying EV-C104, EV-C105, and EV-C109 in respiratory samples. VP4-VP2 and whole-genome sequencing were used to assess genetic diversity and intra-host evolution. **Results**: Five EV-C105 infections were identified primarily in pediatric patients, presenting with a range of clinical features including fever, gastrointestinal symptoms, and cerebellitis. Prolonged EV-C104 and EV-C109 infections were identified in two immunocompromised adults. EV-C104 persisted for over five months and showed evidence of viral genomic changes (intra-host evolution). EV-C109 was detected over a four-month period. Phylogenetic analysis revealed a novel EV-C105 clade (C3) closely related to recent USA strains. EV-C104 genomes aligned with genotype B sequences from the USA and Europe, while EV-C109 sequences were similar to 2014–2015 strains from the Netherlands. **Conclusions**: These findings highlight the emergence, persistence, and genetic evolution of uncommon EV-C types in Maryland, especially among immunocompromised hosts, emphasizing the importance of continued genomic surveillance and clinical correlations.

## 1. Introduction

Enteroviruses (EVs) are a diverse group of RNA viruses belonging to the *Picornaviridae* family, known to cause a wide range of clinical manifestations, from mild respiratory infections to severe systemic diseases such as meningitis, myocarditis, and acute flaccid paralysis [1]. While enteroviruses such as EV-D68 and EV-A71 have been studied due to their association with outbreaks and severe disease, less is known about the uncommon and emerging enterovirus types within the species enterovirus C (EV-C), including EV-C104, EV-C105, and EV-C109 [2,3,4,5,6]. Notably, poliovirus, the most well-recognized EV to cause acute flaccid paralysis (poliomyelitis), belongs to Enterovirus species C [7], highlighting the clinical importance of this group. Emerging EV-C types have been sporadically reported worldwide, often associated with respiratory and neurological disease, but their epidemiology and molecular features remain poorly understood [5,8,9].

EV-C105, first identified in 2010 in the Democratic Republic of Congo, has been linked to acute flaccid paralysis and respiratory infections [9]. Similarly, EV-C109, first reported in Nicaragua in 2008, has been associated with acute respiratory illness in children and has since been detected in Europe and the United States [3,10]. EV-C104, first detected in Switzerland in 2005, has since been infrequently reported [8,11,12,13,14]. Despite their potential clinical significance, the global incidence of these EV-C types appears to be low, likely due to underreporting and the infrequency of EV genomic surveillance [4,5].

In this study, we report EV-C105 detection from samples collected between 2023 and 2024 and tested at the Johns Hopkins Health System. Additionally, we describe prolonged infections with EV-C104 (2018) and EV-C109 (2024) in two immunocompromised patients. By employing next-generation sequencing and phylogenetic analysis, we attempted to elucidate the genetic diversity, evolutionary relationships, and potential clinical significance of these emerging viruses.

## 2. Methods

### 2.1. Ethics and Data Availability

The research was performed with a waiver of consent (the Johns Hopkins IRB protocol IRB00221396, initial approval on 29 October 2019). Genomes are available in GenBank: PV642522-PV642532.

### 2.2. Sample Collection

Remnant respiratory samples including nasopharyngeal swabs and bronchoalveolar lavage (BAL) that tested positive for rhinovirus/enterovirus (RV/EV) using the ePlex respiratory pathogen panels (Roche Diagnostics, Indianapolis, IN, USA) were used for genomic surveillance. Samples had collection dates between 2018 and 2024. For genotyping, nucleic acid was extracted from RV/EV-positive samples, and the VP4-VP2 region was amplified and sequenced as described previously [15,16].

Clinical and demographic data were collected from the electronic medical record system with a bulk query as previously described [17].

### 2.3. EV-C Whole-Genome Sequencing and Sequences Analysis

The Chemagic™ 360 system (Revvity, Walthan, MA, USA) was used for nucleic acid extraction. The LunaScript^®^ RT SuperMix Kit (New England Biolabs (NEB), Ipswish, MA, USA) was used for reverse transcription.

For primary PCR amplification, Q5^®^ High-Fidelity 2× Master Mix was used with primer pairs EV-ABC_80_F and EV-ABC4650_R for the P1 region and EVC-4220-F and C005-R [17,18] for the P2 region (Table S1). The thermal cycling protocol consisted of an initial denaturation at 98 °C for 30 s, followed by 35 cycles of 98 °C for 10 s, 55 °C for 30 s, and 72 °C for 3 min, with a final extension at 72 °C for 10 min. Semi-nested and nested PCR were subsequently performed using primer pairs EV-ABC180_F and EV-ABC4450_R for the P1 region and EVC-4390-F and EVC-7405-R for the P2 region, respectively, while maintaining the same cycling conditions.

PCR products were pooled and purified using NEBNext Sample Purification Beads (NEB) before sequencing. The Native Barcoding (EXP-NBD196) and the NEBNext^®^ ARTIC Library Prep Kits were used for library preparation. Sequencing was performed using the GridION (Oxford Nanopore Technologies, Oxford, England) and R10.4.1 flow cells. The resulting FASTQ files were analyzed using a previously described in-house pipeline (minimum depth cutoff was set at 100×) [19].

For phylogenetic analysis, complete genome sequences were used when available, while VP1 sequences were analyzed when only partial genomes were accessible. These sequences were aligned with all available GenBank references using MAFFT (version 7.450). Maximum likelihood trees were constructed with IQ-TREE3 (version 3.0.0) using 1000 bootstrap replicates, and the optimal nucleotide substitution model was determined with ModelFinder implemented in IQ-TREE3. An in-house script developed in R programming language (https://www.R-project.org/ accessed on 2 May 2024) was used for tree visualization.

To identify novel amino acid and nucleotide substitutions, we used a previously described Python (version 3.11.4). script [19].

## 3. Results

### 3.1. Demographic and Clinical Characteristics of Patients Positive for Uncommon EV-C

Of 3715 samples collected between January 2018 to November 2024 from a total of 3299 unique patients, EV-C105 was identified in 5 samples from 5 patients, EV-C104 in 4 samples from 1 patient, and EV-C109 in 4 samples from 1 patient. The five patients who tested positive for EV-C105 included two males and three females, aged 5 to 31 years and identified as either White (four patients) or Hispanic/Latino (one patient). Clinical presentations were heterogeneous but predominantly involved respiratory and gastrointestinal symptoms. Three patients presented with fever, abdominal pain, vomiting, and cough. One patient developed neurological manifestations including cerebellitis, nystagmus, dizziness, and migraine-like headaches and required intensive care. One patient presented with fever and gastrointestinal symptoms and had a history of asthma, immunosuppression, and hypertension. In total, three of the five patients were hospitalized, and one required ICU-level care. Interestingly, EV-C105 was found in patients from different geographic areas across Maryland, suggesting its widespread regional presence or multiple introductions despite the limited number of cases identified.

Of our screened cohort, we identified a case of a prolonged EV-C104 infection in an adult immunocompromised patient with uncontrolled HIV infection (CD4 count: 4 cells/mm^3^). The patient’s medical history was complicated with chronic hepatitis C, hypertension, hyperlipidemia, chronic pain, and history of substance use. The patient presented with respiratory symptoms, including shortness of breath, productive cough, and fever, along with systemic complaints such as chills, fatigue, and body ache. Gastrointestinal manifestations included diarrhea, nausea, and diffuse abdominal pain. Additional symptoms and signs included oropharyngeal thrush, anorexia, and mild intermittent headaches. Respiratory symptom onset occurred approximately two to three weeks prior to presentation. Despite the initiation of antiretroviral therapy, the patient experienced a persistent respiratory illness, with laboratory testing confirming EV-C104 positivity over a five-month period.

EV-C109 was identified in an adult patient with a history of Epstein–Barr virus (EBV)-positive NK/T-cell lymphoma who had undergone a myeloablative matched unrelated donor allogeneic stem cell transplantation (allo-SCT). The post-transplant course was complicated by chronic graft-versus-host disease (GVHD) affecting the lungs and eyes, resulting in bronchiolitis obliterans syndrome and progressive respiratory failure. The patient developed upper respiratory symptoms and tested positive for RV/EV, which was genotyped as EV-C109. Despite receiving two doses of intravenous immunoglobulin (IVIG), the patient remained persistently PCR-positive for RV/EV.

### 3.2. Phylogenetic Analysis and Viral Evolution

**Figure 1:**
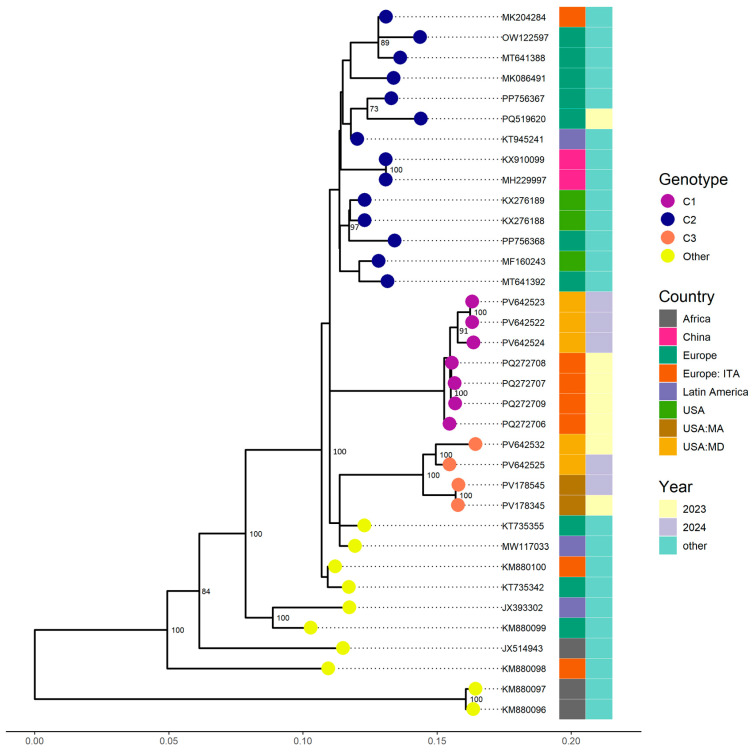
Phylogenetic relationships of EV-C105 strains identified from the Johns Hopkins Health System in 2023 and 2024. Complete and partial genome sequences (VP1) from other countries available from GenBank were included. The phylogenetic tree was constructed using the Maximum Likelihood method in IQ-TREE2 with 1000 bootstrap replicates. Only bootstrap values above 70 were represented on corresponding branches.

Complete genomes were successfully obtained for the four EV-C105 samples collected in 2024, while the 2023 sample yielded a partial genome with 66.1% coverage. Phylogenetic analysis indicated that three of the five sequences belonged to clade C1, clustering with strains identified from Italy in 2023, supported by a bootstrap value of 100% and nucleotide similarity exceeding 98.43% (Figure 1). The remaining two sequences, including the 2023 sample, formed a distinct cluster within clade C3 and were closely related to sequences from Massachusetts (USA) identified in 2023 and 2024, with a bootstrap value of 100% and nucleotide similarity above 97.74%. Notably, clades C1, C2, and C3 exhibit more than 5% sequence divergence from one another, consistent with established thresholds for the classification of well-characterized enteroviruses [20].

For EV-C104, four samples collected at different time points from the same patient were sequenced. Sample 1 (S1) was the initial positive, followed by Sample 2 (S2), collected 1 day later; Sample 3 (S3), collected 108 days after S1; and Sample 4 (S4), collected 160 days after S1. Whole-genome sequences were successfully obtained for three of the samples, while a partial genome with 59.8% coverage was recovered from S2. No additional samples were collected from this patient after S4.

**Figure 2:**
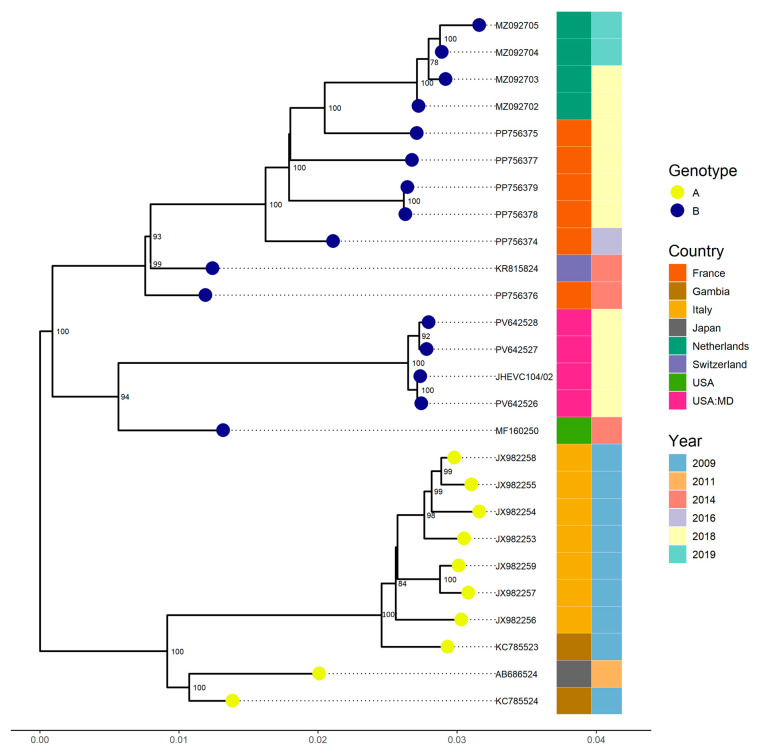
Phylogenetic relationships of four EV-C104 strains identified from samples collected at different time points from the same patient at the Johns Hopkins Health System in 2018. Complete genome sequences from other countries available in GenBank, as well as one partial genome closely related to our strains based on BLAST (version 2.16.0) results were included. The phylogenetic tree was constructed using the Maximum Likelihood method implemented in IQ-TREE2 with 1000 bootstrap replicates. Only bootstrap values greater than 70% are shown on the corresponding branches.

Phylogenetic analysis revealed that all strains belonged to genotype B, with a bootstrap value of 100 and were most closely related to a strain detected in Los Angeles, USA (MF160250, VP4-VP2 region). This strain exhibited a nucleotide similarity exceeding 97.3% with a complete genome sequence from France (PP756376), which had a similarity above 96.6%, both identified in 2014 (Figure 2).

To explore potential intra-host viral evolution, we analyzed single-nucleotide variants (SNVs) over time (Table 1). A total of 23 single-nucleotide variants (SNVs) were identified across the viral genome, 14 of which were shared by both samples (S3 and S4). These included five in the 5′ untranslated region (5′UTR), two each in VP2, VP3, and VP1, one in 2A, two each in 2B and 2C, four in 3D, and one in the 3′ UTR. Of these, six were non-synonymous substitutions, resulting in amino acid changes in both structural and non-structural proteins in each sample. These included E434G in VP3, D675G in VP1, I1618V and I1630T in the 3C protease, and V2133I and N2179D in the 3D polymerase. Notably, the VP1-D675G (D94G) mutation is located within the BC loop.

Regarding EV-C109, four respiratory samples, including one BAL, collected from the same patient at different time points were sequenced. S1 was the initial specimen, followed by S2 which was collected 20 days later; S3 was collected 75 days after S1; and S4, a BAL sample, was collected 82 days after S1. Additionally, five other samples tested positive for RV/EV, including one that was collected 116 days after the initial sample; however, these were not available for sequencing. A subsequent negative RV/EV result was obtained 209 days after the first positive sample.

**Figure 3:**
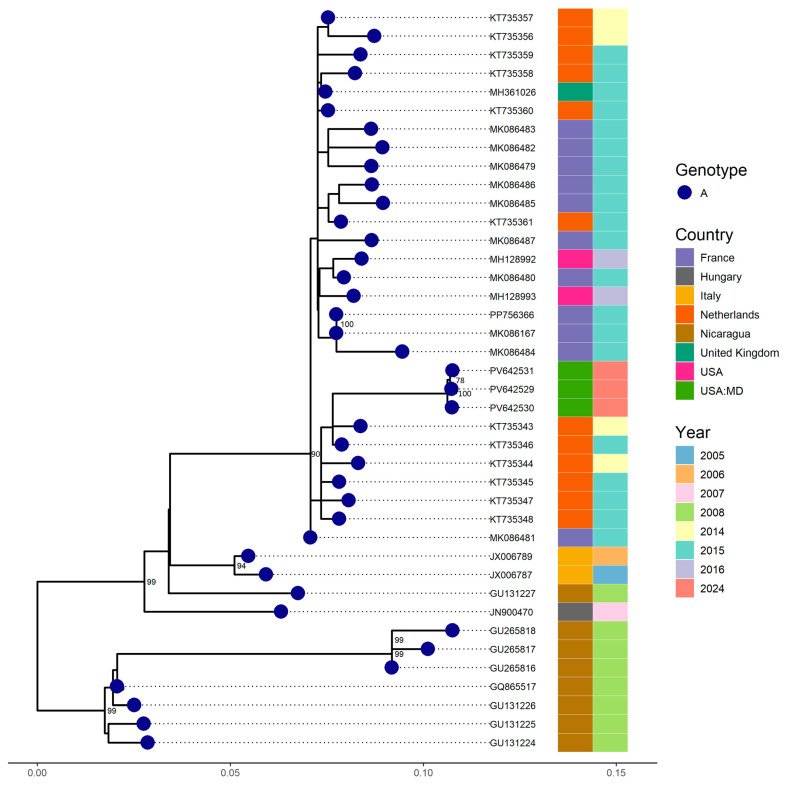
Phylogenetic relationships among four EV-C109 strains identified from samples collected at different time points from the same patient at the Johns Hopkins Health System in 2024. Complete and partial genome (VP1) sequences from other countries retrieved from GenBank were included. A phylogenetic tree was constructed using the Maximum Likelihood method implemented in IQ-TREE2, with 1000 bootstrap replicates. Only bootstrap values above 70% are displayed on the corresponding branches.

Complete genomes were successfully recovered from the three swab samples. Phylogenetic analysis revealed that our EV-C109 strains clustered with sequences (5′UTR region) detected in the Netherlands in 2014 and 2015, showing a nucleotide similarity of approximately 97.92% (Figure 3). However, a comparison with full genomes available in GenBank indicated a nucleotide similarity above 95.6% with strains from Florida (USA) detected in 2018.

Over time, several nucleotide mutations were detected between the initial EV-C109 S1 and the two subsequent samples (S2 and S3) (Table 2). A total of 21 SNVs were identified across the viral genome, distributed among both structural and non-structural regions. These included two SNVs in the 5′UTR, two in VP4, four in VP2, five in VP1, two in 2B, three in 2C, one in 3B, one in 3C, and one in 3D. Among the nucleotide changes, seven resulted in non-synonymous substitutions, leading to amino acid changes across both structural and non-structural proteins. Within the structural proteins, two amino acid changes were identified in VP2 (A190T and A205S), and three in VP1 (S597L, A607S, and S687T). For the non-structural proteins, one mutation was detected in 2C (I1235V) and one in 3D (T1764A). Notably, none of the non-synonymous substitutions were shared between samples.

## 4. Discussion

Of the sporadically reported enterovirus C types, we identified five EV-C105 strains from distinct geographic locations across Maryland between 2023 and 2024, which suggests local viral circulation. This observation aligns with recent EV-C105 detections in several countries, including Spain (5 cases) [4], Italy (6 cases) [5], and Russia (1 case) [21]. Globally, EV-C105 had previously been reported only sporadically between 2010 and 2022 [9,13,22,23,24,25]. The relatively increased recent reporting may be driven by increased global mobility, ongoing viral evolution, increased association with clinical symptomatic disease, or advances in sequencing technologies and surveillance efforts. Notably, the historical low detection rates of EV-C types might have been influenced by the limitations of commonly used cell lines for EV isolations. RD cells, which are widely used, are not optimal for EV-C types, whereas HEp-2C cells have demonstrated improved isolation efficiency [10,26]. Although our study utilized molecular surveillance methods, cell culture limitations may have contributed to the perceived rarity of EV-C105 in earlier studies.

Four of the five EV-C105 infections occurred in children, a trend consistent with prior reports [4,5]. This predominance may reflect immunological naivety, frequent exposure in communal settings, and possibly surveillance biases that prioritize testing in pediatric populations.

One of the EV-C105 cases was associated with cerebellitis, extending the known clinical spectrum of this virus beyond respiratory symptoms. While earlier studies primarily linked EV-C105 to mild respiratory illness [1,9], our observation aligns with rare reports of neurological involvement, including acute flaccid paralysis [4,24]. These findings indicate that further investigation into the neurotropic potential of EV-C105, including viral receptor use, cellular tropism, and host immune interactions is needed.

Phylogenetic analysis revealed the identification of two EV-C105 clades in Maryland: clade C1, identified in 2024 (3/5 sequences), and clade C3, identified in 2023 and 2024 (2/5). Clade C1 sequences were closely related to contemporary strains from Italy and Spain, suggesting recent transcontinental dissemination. Clade C3, newly defined in this study, showed > 5% nucleotide divergence from other clades, consistent with established thresholds for enterovirus classification [4,20]. This finding suggests the emergence of a novel EV-C105 clade circulating in the United States. The observed shift from clade C3 in 2023 to clade C1 in 2024 may reflect a clade replacement dynamic like patterns seen in Spain [4].

We also describe a case of prolonged EV-C104 infection in an immunocompromised patient, characterized by persistent viral detection over five months and evidence of intra-host genomic evolution. Previous studies have identified EV-C104 in both immunocompetent and immunocompromised individuals [6,8,22,27], but data on its evolution during chronic infection is limited.

Phylogenetic analysis confirmed that our EV-C104 strain belonged to genotype B, with >97.3% nucleotide identity to strains from Los Angeles (MF160250) and France (PP756376) identified in 2014, consistent with the global persistence of this lineage [11,13,28]. Intra-host analysis revealed 22 SNVs, including 14 shared across time points and 6 non-synonymous substitutions affecting both structural (e.g., VP1-D675G, VP3-E434G) and non-structural proteins (3C, 3D). These mutations persisted despite the anticipated lack of competent immune pressure, suggesting relaxed purifying selection, a pattern similarly observed in chronic EV infections [8]. Of particular interest, VP1-D675G is located in the BC loop, a region implicated in receptor binding and immune evasion, mirroring other conserved mutations seen in long-term infections [8].

Lastly, we present a case of prolonged EV-C109 infection in an immunocompromised patient. EV-C109 RNA was detected over four months, despite two rounds of IVIG therapy. This finding supports prior reports suggesting EV-C109 can cause persistent infections in immunocompromised individuals [6,8,22,27]. In our EV-C109 case, IVIG was administered, yet viral detection was prolonged. Recent case studies that reported prolonged enterovirus infections showed similar IVIG limitations. Chronic poliovirus infection in an immunodeficient patient and coxsackievirus A1 chronic diarrhea did not resolve after IVIG therapy [29,30]. This highlights the challenges of managing chronic infections caused by EV-C types in immunocompromised hosts.

Phylogenetic analysis revealed close relatedness to strains from the Netherlands (2014–2015) and Florida (2018), with >95.6% nucleotide identity, suggesting global but underrecognized circulation. Whole-genome sequencing of four sequential samples detected 21 nucleotide changes, including seven non-synonymous mutations in VP1, VP2, 2C, and 3D. Notably, VP1 substitutions (S597L, A607S, and S687T) may influence antigenicity or receptor interaction, while a 3D polymerase mutation (T1764A) may affect replication fidelity [31]. These amino acid changes are in both structural and non-structural regions of the genome, which may have functional consequences. Changes in VP1, a key capsid protein, could alter the surface charge or conformation, potentially affecting viral tropism, immune recognition, or receptor binding, even if synonymous. This aligns with previous work showing that conservative or synonymous substitutions in capsid regions can affect the overall protein structure and its interactions [32,33,34,35]. The observed genetic heterogeneity across samples, with limited shared non-synonymous variants, supports a quasispecies model and the presence of intra-host evolution under reduced immune pressure [36].

## 5. Limitations

This study has several limitations. The number of cases per EV-C type, particularly EV-C104 and EV-C109, was small, limiting generalizability. Incomplete genome coverage for some samples prevented comprehensive variant analysis. While several amino acid substitutions were identified, their functional relevance remains speculative in the absence of experimental validation. Samples were collected based on availability and are biased toward the population of patients tested clinically for RV/EV. Despite these constraints, the study contributes valuable insights into the genetic diversity and potential clinical significance of uncommon EV-C types in the USA.

## 6. Conclusions

Our study reports the detection of the rare enterovirus C types EV-C104, EV-C105, and EV-C109 in respiratory samples from Maryland. The identification of a novel EV-C105 clade and the documentation of prolonged EV-C104 and EV-C109 infections in immunocompromised individuals emphasize the need for increased clinical vigilance and ongoing genomic surveillance. Evidence of intra-host viral evolution during chronic infection emphasizes the potential of these viruses for persistence. Importantly, the lack of complete genome sequences for these rare EV-C types in public databases highlights a significant gap in global surveillance efforts and reinforces the need to prioritize full-genome sequencing to better inform diagnostics, treatment strategies, and epidemiological tracking.

## Figures and Tables

**Table 1:** Intra-host variants among EV-C104-positive samples from a single patient. Samples S3 (collected 108 days after S1) and S4 (collected 160 days after S1) were compared to the earliest sample, S1, to assess viral variation over time.

Gene/Region	Nucleotide Substitution (S3)	Nucleotide Substitution (S4)	Amino Acid Substitution
5′UTR	C27T	C27T	
	T29C	T29C	
		T464C	
		A467C	
	T576C		
VP2	C1173A		
		G1383A	
VP3	A2017G	A2017G	E434G
		G2045A	
VP1	A2740G	A2740G	D675G
	G2747A	G2747A	
2A	C3512T	C3512T	
2B	G3878A	G3878A	
		C3887T	
2C	C4122T		
	G4703A		
3C	A5568G	A5568G	I1618V
	T5605C	T5605C	I1630T
3D	G6632A	G6632A	
	A6905G	A6905G	
	G7113A	G7113A	V2133I
	A7251G	A7251G	N2179D
3′UTR	T7393 Del	T7393 Del	

**Table 2:** Intra-host variants among EV-C109-positive samples from a single patient. Samples S2 (collected 20 days after S1) and S3 (collected at 75 days after S1) were compared to the earliest sample, S1, to assess viral variation over time.

Gene/Region	Nucleotide Substitution (S2)	Nucleotide Substitution (S3)	Amino Acid Substitution
5′UTR	T114C	T114C	
		T167C	
VP4	T691C		
VP2	T931C		
		G1166A	A190T
	T1174C		
	G1211T		A205S
VP1		C2388T	A607S
		G2417T	S687T
	T2579C		
		C2593T	
	T2657A		S597L
	G2779A		
2B	T3739C		
	C3895T	C3895T	
2C	A4301G		I1235V
	T4396C		
	C4804T		
3B	C5227T		
3C	T5533C	T5533C	
3D	A5888G		T1764A

## Data Availability

Data is contained within the article.

## Supplementary Materials

The following supporting information can be downloaded at: https://www.mdpi.com/article/10.3390/v17091183/s1, Table S1: Primers used for whole-genome amplification of the EV-C.

## Abbreviations

The following abbreviations are used in this manuscript:

EV	Enteroviruses
EV-C	Enterovirus C
RV/EV	Rhinovirus/enterovirus
BAL	Bronchoalveolar lavage
EBV	Epstein–Barr virus
allo-SCT	Allogeneic stem cell transplantation
GVHD	Graft-versus-host disease
IVIG	Intravenous immunoglobulin
SNVs	Single-nucleotide variants
