# Supplementary material for: Molecular Characterization of Emerging and Uncommon Enteroviruses C104, C105, and C109 in Respiratory Samples from Maryland, USA, 2018–2024
Source: Viruses. 2025 Aug 29;17(9):1183. doi: 10.3390/v17091183 (PMC12474150; doi:10.3390/v17091183)
Supplement: Supplementary file 1 [file viruses-17-01183-s001.zip › viruses-3820201-supplementary.pdf]

Table S1. Primers used for whole-genome amplification of the EV-C.

Name	Sequences	Size (nt)	Reference position
EV-ABC80-F	ACCYTTGTGCGCCTGTTTTAT	21	69-89
EV-ABC4650_R	CGGTGTTTGSTCTTGAACT	21	4474-4456
EVC-4220-F	GARGCNTGYAAYGCNGCNAARG	22	4146-4167
C005-R	CCGAATYAAARRAAAATTTACCC	23	7430-7408
EV-ABC180_F	CAAGCACTTCTGTYTTCCCG	20	162-181
EVC-4390-F	GYCCNAGYCARGAASAHCARG	21	4318-4338
EVC-7405-R	GACTGAGGTAGGGTTACTAAA	21	7380-7360

Reference = V01148.1 (poliovirus type 1 (Mahoney strain).
